# Ebola exposure, illness experience, and Ebola antibody prevalence in international responders to the West African Ebola epidemic 2014–2016: A cross-sectional study

**DOI:** 10.1371/journal.pmed.1002300

**Published:** 2017-05-16

**Authors:** Catherine F. Houlihan, Catherine R. McGowan, Steve Dicks, Marc Baguelin, David A. J. Moore, David Mabey, Chrissy h. Roberts, Alex Kumar, Dhan Samuel, Richard Tedder, Judith R. Glynn

**Affiliations:** 1 Faculty of Infectious and Tropical Diseases, London School of Hygiene & Tropical Medicine, London, United Kingdom; 2 Faculty of Medical Sciences, University College London, London, United Kingdom; 3 Faculty of Public Health and Policy, London School of Hygiene & Tropical Medicine, London, United Kingdom; 4 Humanitarian Public Health Technical Unit, Save the Children UK, London, United Kingdom; 5 Transfusion Microbiology, National Health Service Blood and Transplant, London, United Kingdom; 6 NHSBT/PHE Blood Borne Virus Unit, Serology Development Unit, Public Health England, London, United Kingdom; 7 Centre of Infectious Disease Surveillance and Control, Public Health England, London, United Kingdom; 8 Faculty of Epidemiology and Population Health, London School of Hygiene & Tropical Medicine, London, United Kingdom; 9 Department of Infection and Tropical Medicine, University Hospitals of Leicester NHS Trust, Leicester, United Kingdom; Groote Schuur Hospital, SOUTH AFRICA

## Abstract

**Background:**

Healthcare and other front-line workers are at particular risk of infection with Ebola virus (EBOV). Despite the large-scale deployment of international responders, few cases of Ebola virus disease have been diagnosed in this group. Since asymptomatic or pauci-symptomatic infection has been described, it is plausible that infections have occurred in healthcare workers but have escaped being diagnosed. We aimed to assess the prevalence of asymptomatic or pauci-symptomatic infection, and of exposure events, among returned responders to the West African Ebola epidemic 2014–2016.

**Methods and findings:**

We used snowball sampling to identify responders who had returned to the UK or Ireland, and used an online consent and questionnaire to determine their exposure to EBOV and their experience of illness. Oral fluid collection devices were sent and returned by post, and samples were tested using an EBOV IgG capture assay that detects IgG to Ebola glycoprotein. Blood was collected from returnees with reactive samples for further testing. Unexposed UK controls were also recruited.

In all, 300 individuals consented, of whom 268 (89.3%) returned an oral fluid sample (OFS). The majority had worked in Sierra Leone in clinical, laboratory, research, and other roles. Fifty-three UK controls consented and provided samples using the same method.

Of the returnees, 47 (17.5%) reported that they had had a possible EBOV exposure. Based on their free-text descriptions, using a published risk assessment method, we classified 43 (16%) as having had incidents with risk of Ebola transmission, including five intermediate-risk and one high-risk exposure. Of the returnees, 57 (21%) reported a febrile or diarrhoeal illness in West Africa or within 1 mo of return, of whom 40 (70%) were not tested at the time for EBOV infection.

Of the 268 OFSs, 266 were unreactive. Two returnees, who did not experience an illness in West Africa or on return, had OFSs that were reactive on the EBOV IgG capture assay, with similar results on plasma. One individual had no further positive test results; the other had a positive result on a double-antigen bridging assay but not on a competitive assay or on an indirect EBOV IgG ELISA. All 53 controls had non-reactive OFSs. While the participants were not a random sample of returnees, the number participating was high.

**Conclusions:**

This is the first study, to our knowledge, of the prevalence of EBOV infection in international responders. More than 99% had clear negative results. Sera from two individuals had discordant results on the different assays; both were negative on the competitive assay, suggesting that prior infection was unlikely. The finding that a significant proportion experienced “near miss” exposure events, and that most of those who experienced symptoms did not get tested for EBOV at the time, suggests a need to review and standardise protocols for the management of possible exposure to EBOV, and for the management of illness, across organisations that deploy staff to outbreaks.

## Abstract 2

In a cross-sectional study, Catherine Frances Houlihan and colleagues surveyed international responders to the West African Ebola crisis, after they returned to the UK or Ireland, to analyze their experience of risk.

## Author summary

**Why was this study done?:**

- A large number of international volunteers worked in West Africa during the largest Ebola outbreak ever seen, but only a small number of international staff were diagnosed with Ebola virus disease. Since asymptomatic or unrecognised infections can occur, it is possible that some infections in international responders to the epidemic were missed.

**What did the researchers do and find?:**

- We carried out an online survey and antibody testing of oral fluid samples from 268 individuals who returned to the UK and Ireland after working in West Africa during the Ebola outbreak. A high number of near miss events were reported, and less than a third of individuals who experienced illness whilst in West Africa or soon after return were tested for Ebola virus while they were unwell. Of the 268 who were tested for antibodies, two had reactive test results, but these were not reactive on further testing.

**What do these findings mean?:**

- Although our study could not reach every individual who returned to the UK after working in West Africa during the Ebola outbreak, we demonstrated that asymptomatic or pauci-symptomatic Ebola virus infection was rare in international healthcare and other workers in our study who responded to the epidemic in West Africa.
- The descriptions of near miss events and the finding that many of those who experienced illness were not tested at the time suggest that protocols for the management of possible exposure to Ebola virus and for the management of illness should be reviewed and standardised across organisations that deploy staff to outbreaks.

## Introduction

In Ebola virus disease (EVD) epidemics, healthcare workers (HCWs) are disproportionately affected. In the West African 2014–2016 Ebola epidemic, the percentage of HCWs infected was 1.45% in Guinea (compared to 0.02% in the general population), 8.07% in Liberia (compared to 0.11%), and 6.85% in Sierra Leone (compared to 0.06%) [1]. The outbreak occurred in countries that have some of the lowest proportions of HCWs per head of population in the world [2]. This fact—coupled with the rapid spread of the epidemic, the high case fatality rate [3–7], and serious concerns raised by the international community—motivated the World Health Organization (WHO) to declare the outbreak as a “public health emergency of international concern” and resulted in the escalated deployment of international emergency medical teams (EMTs) to West Africa (primarily to Guinea, Liberia, and Sierra Leone) beginning in late 2014 [8]. These EMTs included water, sanitation, and hygiene (WASH) and laboratory workers, epidemiologists, engineers, logisticians, and operations staff as well as clinicians. To date, WHO reports that 40 organisations from 19 countries sent EMTs to West Africa in response to the 2014–2016 Ebola outbreak [9]; over 3,000 individuals were deployed to West Africa by the US Centers for Disease Control and Prevention alone [10]. Despite this large-scale deployment, few cases of EVD were diagnosed among EMT workers: 24 were treated in Europe or the US [8].

In Kikwit, Democratic Republic of the Congo, in an Ebola outbreak in 1995, 8/402 (2%) HCWs in the outbreak area had positive serology in the absence of diagnosed EVD. Among asymptomatic household contacts of cases, the prevalence of positive serology has ranged from 1% to 46% in different studies using different assays [11–16]. More recently, serology positive for Ebola virus (EBOV) infection was found in 12.0% (11/92) of household contacts with symptoms in Sierra Leone, and 2.6% (10/389) of those with no symptoms, using a highly sensitive and specific IgG assay [17]. A recent meta-analysis estimated that 27% of all infections are asymptomatic [18], with similar findings from a “hot spot” in Sierra Leone [19]. It is therefore plausible that asymptomatic or pauci-symptomatic infections have occurred in HCWs but have escaped being diagnosed.

We describe the prevalence of antibodies to EBOV in oral fluid samples (OFSs) from individuals who returned to the UK or Ireland after responding to the West African EVD epidemic, as well as potential exposures to EBOV and experience of illness in West Africa or within 1 mo of return.

## Methods

### Recruitment

Invitations to participate were sent to individuals known to the authors, and through organisations supporting EMT deployment involving UK-based staff, including non-governmental organisations (NGOs), UK government-affiliated institutions, and the London School of Hygiene & Tropical Medicine (LSHTM). A request was embedded in both the email and at the end of the online questionnaire to forward the email to other eligible persons—“snowball sampling” [20]. The email described the purpose of the study and the methods of data collection, and included a link to an online information sheet, consent form, and questionnaire.

The study was also advertised via posters and cards at selected meetings that eligible individuals were likely to attend (with permission from the meeting organisers). The study information and a link to the survey were also distributed via social media platforms (Facebook and Twitter).

UK control individuals with no known exposure to filoviruses were also recruited at LSHTM and through personal contacts.

An online consent procedure was employed for this study and consisted of an information sheet and consent form included at the beginning of the online survey (see S2 Text). The survey was accessible only after consent and was opened on 16 December 2015 and closed on 16 June 2016. Ethics approval was obtained from the LSHTM Research Ethics Committee (approval #9475). All data were stored on the LSHTM secure data server and were subject to file access logging.

### Eligibility criteria

Eligible participants were ≥18 y of age, had travelled to West Africa during the 2014–2016 EVD outbreak as part of the response, had never tested positive for EBOV by PCR, and had never received a filovirus vaccine.

UK controls were ineligible if they were <18 y of age, had taken immunosuppressant medication in the past year, had spent a prolonged period (>1 mo) in any country with EBOV transmission, had ever had contact with patients with exposure to or infected with filoviruses, had worked with filoviruses in a laboratory setting, or had participated in a filovirus vaccine study.

### Questionnaire

The questionnaire asked for basic demographic information and information about returnees’ deployment to West Africa, and any occupational risks or potential exposures (see S2 Text). Returnees were asked if they had experienced a febrile illness during their deployment or within 1 mo of return. For those using personal protective equipment (PPE), details were asked about the methods used for doffing. A broad definition of PPE was used but included a protective suit (with or without hood) with gloves (single or multiple pairs) and with eye protection, with or without respiratory protection.

### Oral fluid collection kit

Oral fluid collection devices (Oracol S10, Malvern Medical Developments) were sent via the UK postal service to consenting eligible individuals. Returnees collected the OFS using a sponge massaged along the gum line for 90 s. Returnees could follow a pictorial instruction leaflet or follow a link to a video demonstration (http://tinyurl.com/lshtmsample).

In order to minimise potential delays, a request to post specimens within 24 h of collection was included. Specimens were stored at −80°C°C within 24 h of receipt at LSHTM. Collection continued until 300 returnees had consented to participate and recruitment was slowing. All analyses were done after the end of data collection.

### Sample testing

The OFSs were tested for EBOV glycoprotein IgG using an IgG capture assay. Oral fluid EBOV glycoprotein IgG levels have been shown to be closely correlated with plasma IgG level (*R*^2^ = 0.78) [14]. In a field study in Sierra Leone, the assay had a sensitivity of 96% (93/97 recovered patients with PCR-confirmed EVD tested positive) and a specificity of 100% (339/339 controls tested negative) [17]. Testing was carried out in two batches on 12 May 2016 and 22 June 2016, and samples received after 22 June 2016 were destroyed. Specimens were defrosted and extracted with 1 ml of transport medium. One hundred microlitres of extract was incubated in individual wells coated with anti-human IgG, along with two positive and two negative controls, before being washed. A conjugate of recombinant EBOV Zaire (Mayinga strain) glycoprotein antigen (rGPδTM, cat. 0501–016, IBT Bioservices) coupled with horseradish peroxidase antigen was added, further incubated, then washed. Bound conjugate was detected using TMB substrate, and optical density (OD) measurements were made on an ELx808 Ultra Microplate Reader (Biotek Instruments) and analysed using Kineticalc software for Windows (BioTek). The cutoff for each plate was taken as the mean of the negative controls in that plate plus 0.1 OD, derived from a standard curve method applied to samples reported elsewhere [21,22]. In order for a plate to be valid, the OD of the positive control had to be >1.0, and that of the negative control <0.1. Normalised ODs (NODs) were calculated using the ratio of the test OD to the defined test cutoff. A NOD > 1 was considered reactive.

Those with reactive OFSs were contacted, and 5 ml of blood was collected. Plasma samples were tested at Public Health England (PHE) for EBOV RNA using a real-time pan-Ebola PCR [23], and for EBOV antibodies using the IgG capture assay and, additionally, a competitive ELISA that included a labelled murine monoclonal 4G7 antibody and a double antigen bridging assay (DABA), both of which use the same recombinant glycoprotein as the IgG capture assay. Finally, plasma samples were sent to the Jenner Institute in Oxford, where they were tested using an ELISA that detects IgG to glycoprotein of EBOV Mayinga strain (amino-acids 1–649 of GenBank protein AHX24649.1), an in-house assay used to measure Ebola vaccine response [24]. Because of a limited supply of recombinant glycoprotein antigen and monoclonal antibody at PHE, no negative samples were tested using these second-line tests. The positive control in all PHE tests was a 1:200 dilution of serum from a recovered UK Ebola patient. No internal quality control was incorporated to verify the presence of adequate total immunoglobulin in the OFSs, but all samples were checked visually to ensure that the un-extracted swabs were not dry.

### Statistical methods

Analyses were performed using STATA 14 (StataCorp).

The risk of infection in individuals was categorised as very low, low, intermediate, or high, using the stratification devised by Jacobs et al. [25], applied to the survey free-text answers from returnees, with modification to include breaches in PPE. Using this system, being physically close (<1 m) to an individual with confirmed EVD whilst not wearing PPE, or with a breach in the PPE, constituted very low risk if there was no direct physical contact, low risk if there was direct physical contact, and intermediate risk if the patient had vomiting, diarrhoea, or bleeding. Contact with body fluids, either directly from a patient or with the environment, was considered intermediate risk if there was no exposure onto broken skin or mucous membranes or high risk if there was contact with these areas. In transcutaneous exposure (such as a needle stick injury), risk was considered high if the sharp was a freshly used hollow-bore needle and high if the sharp object had been contaminated by contact with a confirmed EVD patient or body fluids. The risk from a percutaneous injury was considered intermediate if the sharp was not known to have been directly contaminated [25].

Factors associated with risk of exposure, experiencing a febrile/diarrhoeal illness in West Africa or within 1 mo of return, and being tested for EBOV during such illness were determined using logistic regression. Factors reaching *p* < 0.1 in the univariate analysis were included in a multivariable model. Tests for trend were calculated using the likelihood ratio test to compare a linear model with a categorical model.

## Results

### Participants

It was not possible to estimate the number who received the survey via email, but online consent was received from 300 individuals, all of whom completed the survey; of these, 268 (89.3%) returned an OFS and were included in the analysis. Fifty-three UK control participants consented and provided an OFS using the same procedures.

### Descriptive data

The median age for those who returned a sample was slightly older than for those who did not (39 versus 33 y, respectively; *p* = 0.07); all other characteristics were similar between the groups. Of the 268 returnees who returned samples, 152 (57%) were female; the median age was 36 y (interquartile range [IQR] 30–45). The majority had most recently worked in Sierra Leone (253, 94%) and had worked in PPE (230, 86%) (Table 1). The median age of the controls was 35 y (IQR 31–40), and 35 (66%) were female.

**Table 1:** Characteristics of 268 individuals who travelled to West Africa in response to the 2014–2016 Ebola epidemic.

Characteristic	Number (percent) or median (IQR)
**Demographics**	
Female	152 (56.7%)
Age (years)	36 (30–45)
**Country most recently worked in**	
Sierra Leone	253 (94.4%)
Liberia	12 (4.5%)
Guinea	3 (1.1%)
**Roles undertaken (returnees could select more than one)**	
Laboratory	95
Physician	70
Nurse	54
Research	37
Management/operations	28
Trainer	23
Epidemiologist	19
Community engagement/tracing	18
WASH staff	11
Finance	3
Engineer	3
Pharmacist	2
Social worker/burial team/information technology/journalist/visitor/logistician/nutritionist^1^	7
**Worked in PPE (laboratory or clinical)**	233 (86.9%)
**Time in country (days)**	30 (20–40)
**Previous experience**	
Laboratory work with filoviruses	11
Haemorrhagic fever outbreak	7
Both lab and outbreak work	3

^1^One returnee reported each of these roles.

IQR, interquartile range; PPE, personal protective equipment; WASH, water, sanitation, and hygiene.

### Contact and exposure

**Fig 1:**
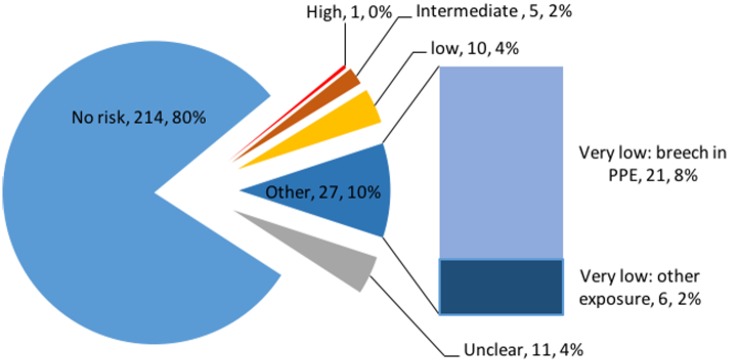
Risk of Ebola virus disease transmission in 268 individuals who travelled to West Africa in response to the 2014–2016 Ebola epidemic. PPE, personal protective equipment.

Possible exposure was ascertained through several different questions, and free-text responses were encouraged to describe incidents more fully. Twenty-one (8%) individuals reported having had direct physical contact with patients with confirmed or suspected EVD whilst not wearing PPE. Fifty-five (21%) answered “yes” (47) or “unsure” (8) when asked whether they had had a significant exposure or a “near miss” event. Using the free-text descriptions, exposures were allocated a level of risk of transmission (Box 1). In all, 43 (16%) individuals had potential exposures: 27 (10%) very low risk, 10 (4%) low risk, five (2%) intermediate risk, and one high risk (Fig 1).

**Box 1. Examples of risk classification, and associated free-text descriptions from the survey, among 268 individuals who travelled to West Africa in response to the 2014–2016 Ebola epidemic**

**No or unclear risk:** direct contact with local population with no known illness; torn outer glove, inner glove intact.

“Children and some adults would approach you unexpectedly and grab your arm etc. Contact with locals, they did not look ill as were mainly walking on streets or playing”

“Torn glove in isolator but remaining gloves intact and no fluid contact”

**Very low risk:** living or working with someone with undiagnosed EVD but no direct physical contact; breaches in PPE with no visible contamination or broken skin exposure

“Torn suit when caring for a sick woman who was fitting”

“Facemask dislodged”

“Person I was staying in same accommodation with became infected”

**Low risk:** direct physical contact with patient who does not have vomiting, diarrhoea, or bleeding.

“Contact with subsequently confirmed infected Medic…contact was symptomatic but ‘dry’”

“A local colleague working at our ETU [Ebola treatment unit] came to work while symptomatic for approximately 5 days without informing us, he tested positive and died from EVD a week later”

**Intermediate risk:** direct physical contact with a patient with diarrhoea, vomiting, or bleeding; direct contact with bodily fluids from a patient with EVD.

“Cut hand on artesunate vial (through gloves) whilst in red-zone”

“Being vomited on while wearing just gloves”

“Touched mask with blood on gloves (could not breathe—had to pull mask from face)”

**High risk:** exposure of mucous membranes or broken skin to bodily fluids from an EVD patient, or sharp injury from a needle/other used on an EVD patient (not freshly used or not hollow-bore needle).

“Sharp injury with a broken vial of medication inside the red zone with dirty and contaminated gloves”

Table 2 shows the factors associated with reporting any transmission risk compared to reporting no/unclear risk. The amount of time spent in PPE in the laboratory or the clinical red zone was associated with increased odds of experiencing an EBOV transmission risk (*p* for trend = 0.02, excluding those not wearing PPE). Returnees were more likely to report an exposure risk if they had been performing a clinical role (29% of 123) compared to a laboratory role (3% of 90) (odds ratio 13.4 [95% CI 3.8–47.0] after adjusting for time in PPE) (Table 2). After adjusting for role and days in PPE, no other factors were associated with risk. The different methods of PPE removal were associated with the role, so were not independently associated with risk.

**Table 2:** Factors associated with risk of Ebola virus disease transmission in 268 returnees who worked in West Africa during the 2014–2016 Ebola outbreak.

Factor	Number (percent)	Risk of EVD transmission, *n* (percent)	Unadjusted OR (95% CI)	Adjusted OR^1^ (95% CI)	*p-*Value
**Sex**					
Female	152 (56.7)	20 (13.2)	1		
Male	116 (43.3)	23 (19.8)	1.6 (0.8–3.1)		
**Age group (years)**					
19–30	73 (27.2)	8 (11.0)	1		
31–40	99 (36.9)	19 (19.2)	1.9 (0.8–4.7)		
41–50	45 (16.8)	8 (17.8)	1.8 (0.6–5.1)		
≥51	51 (19.0)	8 (16.7)	1.5 (0.5–4.3)		
**Country**					
Sierra Leone	253 (94.4)	41 (16.2)	1		
Guinea/Liberia	15 (5.6%)	2 (13.3)	0.8 (0.2–3.7)		
**Facility**^2^					
Facility 1	72 (26.9)	16 (22.2)	1		
Facility 2	67 (25.0)	7 (10.5)	0.4 (0.2–1.1)		
Facility 3	53 (19.8)	3 (5.7)	0.2 (0.1–0.8)		
Facility 4	13 (4.9)	6 (46.2)	3.0 (0.9–10.2)		
Other	63 (23.5)	11 (17.5)	0.7 (0.3–1.7)		
**Role**^3^					<0.01
Laboratory work	90 (33.6)	3 (3.3)	1	1	
Clinical work	123 (45.9)	35 (28.5)	11.5 (3.5–38.9)	13.4 (3.8–47.0)	
Other role^4^	55 (20.5)	5 (9.1)	2.9 (0.7–12.7)	8.7 (1.6–47.4)	
**Days in PPE**^5^					0.02; test for trend, 0.02^6^
1–25	77 (28.7)	13 (16.9)	1	1	
26–35	83 (31.0)	9 (10.8)	0.6 (0.2–1.5)	1.3 (0.5–3.6)	
36–60	38 (14.2)	9 (23.7)	1.5 (0.6–4.0)	2.3 (0.8–6.4)	
>60	22 (8.2)	9 (41.0)	3.4 (1.2–9.6)	4.7 (1.5–14.8)	
None	48 (17.9)	3 (6.3)	0.3 (0.1–0.4)	0.3 (0.1–1.4)	
**Previous viral haemorrhagic fever work**					
No previous work	247 (92.2)	41 (16.6)	1		
Previous work	21 (7.8)	2 (9.5)	0.5 (0.1–2.3)		
**PPE removal—chlorine spray**^7^					
No spray	98 (36.6)	7 (7.1)	1		
Chlorine spray	132 (49.3)	33 (25.0)	4.3 (1.8–10.3)		
Not applicable	38 (14.2)	3 (7.9)	1.1 (0.3–4.6)		
**PPE removal—assistance**^7^					
No assistance	108 (40.3)	13 (12.0)	1		
Assistance	122 (45.5)	27 (22.1)	2.1 (1.0–4.3)		
Not applicable	38 (14.2)	3 (7.9)	0.6 (0.2–2.3)		

^1^Only days in PPE and laboratory work were included in the final model.

^2^Facility is the Ebola treatment centre or holding facility at which the returnee was predominantly based. Facilities 1–4 were the most commonly reported facilities.

^3^It was possible to select more than one role. Where individuals selected both laboratory and clinical red zone (*n* = 3), they were allocated to the role in which they reported more time in subsequent questions. “Clinical” includes nurses, doctors, and paramedics.

^4^Other roles include those listed in Table 1.

^5^Thirty-eight individuals did not spend any time in PPE. An additional ten did not provide information on the duration of time in PPE.

^6^Excluding those who did not wear PPE.

^7^On exiting the clinical red zone or laboratory. PPE removal was not included in the final model since method of removal was almost collinear with role.

CI, confidence interval; EVD, Ebola virus disease; OR, odds ratio; PPE, personal protective equipment.

Over one-third of returnees reported contact with recovered EVD patients (98/268, 37%). Most of these (57/98, 58%) were direct physical contact (handshaking or embracing) with patients at the time of discharge from an Ebola treatment centre (ETC), which was encouraged. Others described living or working with recovered patients (9, 3%) or contact within a follow-up clinic setting (14, 5%), or they reported contact but did not provide a clear description (18, 7%). These contacts were not included as transmission risks.

### Self-reported illness and testing

Among 268 returnees who were never known to have had EVD, 57 (21%) experienced a febrile/diarrhoeal illness either in West Africa or within 1 mo of return. Table 3 shows the factors associated with illness and with being tested for EBOV if the returnee experienced diarrhoea and/or fever.

**Table 3:** Factors associated with experiencing an illness in 268 returnees from the West African Ebola 2014–2016 outbreak, and factors associated with having been tested for Ebola virus in 57 who experienced an illness.

Factor	Number (percent)	Experience of illness, *n* (percent)	OR (95% CI)	*p-*Value	Tested for Ebola virus, *n* (percent)	OR (95% CI)	*p-*Value
**Sex**							
Female	152 (56.7)	32 (21.1)	1		10 (31.3)	1	
Male	116 (43.3)	25 (21.6)	1.0 (0.6–1.9)		7 (28.0)	0.8 (0.3–2.7)	
**Age group (years)**							
19–30	73 (27.2)	20 (27.4)	1		6 (30.0)	1	
31–40	99 (36.9)	21 (21.2)	0.7 (0.4–1.4)		6 (28.6)	0.9 (0.2–3.6)	
41–50	45 (16.8)	8 (17.8)	0.6 (0.2–1.4)		1 (12.5)	0.3 (0.0–3.3)	
≥51	51 (19.0)	8 (15.7)	0.5 (0.2–1.2)		4 (50.0)	2.3 (0.4–12.6)	
**Role**^1^				<0.01			
Laboratory work	90 (33.6)	11 (12.2)	1		4 (34.4)	1	
Clinical work	123 (45.9)	35 (28.5)	2.9 (1.4–6.0)		12 (34.3)	0.9 (0.2–3.8)	
Other role^2^	55 (20.5)	11 (20.0)	1.8 (0.7–4.5)		1 (9.1)	0.2 (0.0–1.9)	
**Risk of EVD**							
No risk/unclear	225 (84.0)	47 (20.9)	1		12 (25.5)	1	
Risk	43 (16.0)	10 (23.3)	1. 1 (0.5–2.5)		5 (50.0)	2.9 (0.7–11.9)	
**Facility**^3^							
Facility 1	72 (26.9)	14 (19.4)	1		7 (50.0)	1	
Facility 2	67 (25.0)	18 (26.9)	1.5 (0.7–3.4)		5 (27.8)	0.4 (0.1–1.7)	
Facility 3	53 (19.8)	8 (15.1)	0.7 (0.3–1.9)		1 (12.5)	0.1 (0.0–1.5)	
Facility 4	13 (4.9)	7 (53.9)	4.8 (1.4–16.6)		3 (42.9)	0.8 (0.1–4.7)	
Other	63 (23.5)	10 (15.9)	0.8 (0.3–1.9)		1 (10.0)	0.1 (0.0–1.1)	
**Illness outcome**							<0.01
Illness in country	21 (36.8)				1 (4.8)	1	
Illness in UK	17 (29.8)				11 (64.7)	36.7 (3.9–244.8)	
Other cause	19 (33.3)				5 (26.3)	7.1 (0.8–68.0)	

^1^It was possible to select more than one role. Where individuals selected both laboratory and clinical red zone (*n* = 3), they were allocated to the role in which they reported more time in subsequent questions. “Clinical” includes nurses, doctors, and paramedics.

^2^Other roles include those listed in Table 1.

^3^“Facility” is the Ebola treatment centre or holding facility at which the returnee was predominantly based. Facilities 1–4 were the most commonly reported facilities. Facility was not included in the adjusted model since it was almost collinear with role.

CI, confidence interval; EVD, Ebola virus disease; OR, odds ratio.

Clinical staff were the most likely to have experienced an illness; 29% (35 of 123) compared to 20% (11 of 55) of “other” staff and 12% (11 of 90) of laboratory staff (*p* < 0.01) (Table 3). After adjusting for role, no other factors were associated with illness.

Amongst 57 returnees who experienced a febrile/diarrhoeal illness, 30% (17 returnees) were tested for EBOV infection at the time. One of 21 (5%) who fell ill in country had an EBOV PCR, compared to 11 of 17 (65%) who fell ill on return (odds ratio 36.7 [95% CI 3.9–244.8]). Of those who were ill and were classified as having no or unclear risk (47 returnees), 12 (25%) were tested, compared to five of the ten (50%) with some risk. The individual with a high-risk exposure did not describe an illness and was not tested. Of the five with intermediate-risk exposures, three were ill, and two of those were tested.

### Laboratory results

**Fig 2:**
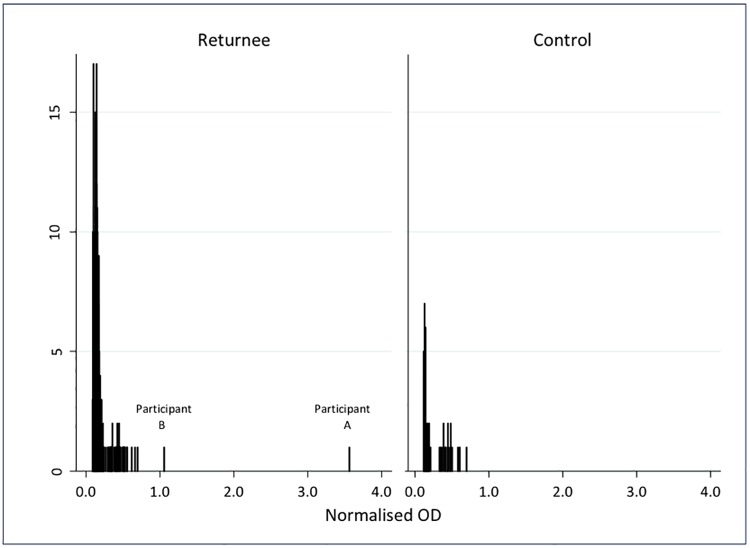
Normalised optical density results for IgG in oral fluid samples from 268 individuals who travelled to West Africa in response to the 2014–2016 Ebola epidemic, and 53 UK controls.

The frequency of NOD values for the 268 OFSs are shown in Fig 2, alongside those from 53 controls. All the samples from UK controls and all but two of the samples from returnees were well below the cutoff (maximum NOD 0.7). The two samples that were reactive on the first test had similar results on re-testing. Plasma from these individuals was tested further, as described below.

### Participant A

Participant A was a female laboratory worker who spent a total of 30 d in an Ebola laboratory in West Africa. She did not describe a significant exposure or febrile illness, and was not tested with PCR for EBOV infection at any point during her deployment or on return.

The OFS was reactive on three tests, with NOD 3.55, 3.27, and 3.12. In plasma, the IgG capture assay was reactive twice (NOD 4.5 and 5.4), the DABA was reactive twice (NOD 2.6 and 3.2), but the competitive assay was unreactive twice (NOD 0.44 and 0.49), and the in-house indirect ELISA test at the Jenner Institute was unreactive. The EBOV PCR on plasma was negative.

### Participant B

Participant B was a female physician who spent 40 d in the clinical red zone, did not describe a significant exposure or any illness, and was not PCR tested for EBOV infection during her deployment or on return. The OFS showed a low level of reactivity on the IgG capture assay (NOD 1.05, 1.26, and 0.88). Similar low-level reactivity was seen in the IgG capture assay of plasma (NOD 1.40 and 1.24). The plasma was unreactive on DABA (NOD 0.43 and 0.41), on the competitive assay (NOD 0.47 and 0.45), and on the Jenner Institute indirect ELISA. The EBOV PCR on plasma was negative.

## Discussion

This is the first examination to our knowledge of possible undiagnosed infections with EBOV in international HCWs and other responders working during the West African 2014–2016 Ebola outbreak. More than 99% had no reactivity on the screening test, making undetected infection, at most, a very rare event. The two individuals with reactive tests for IgG to EBOV glycoprotein antigen reported no significant exposure. One of these individuals demonstrated a level of reactivity around the cutoff, illustrating non-specific reactivity, a possible finding in serological diagnostics [26]. The other individual had results that were reactive in the IgG capture assay at a level seen in recovered EVD patients [17], and reactive in a DABA, but not in a competitive assay and not in a separate indirect IgG ELISA. Both the capture assay and the DABA employ exactly the same recombinant antigen (EBOV glycoprotein); therefore, concordant positivity on both could be expected. The competitive assay, however, uses the monoclonal antibody 4G7, a constituent of the proposed therapy for EVD ZMAb [27], which competes with the participant’s own antibody for binding on the same recombinant EBOV glycoprotein and is expected to be inherently more specific. The result in this returnee could be explained through cross-reactivity, the presence of an antibody that binds to a single epitope on the EBOV glycoprotein used in the PHE IgG capture assay that was not present on the EBOV glycoprotein used in the Jenner Institute IgG indirect ELISA and was not produced in response to EBOV infection. Alternatively, the returnee could have been infected with a small inoculum and generated a minimal subsequent memory B cell response resulting in either a low-avidity antibody or one with a limited epitope profile. A low-titre antibody does not seem an adequate explanation since the geometric mean titres of antibody in asymptomatic undiagnosed household contacts of cases of EVD in Sierra Leone were not significantly different from those of recovered patients [17]. Since the returnee was not ever symptomatic, displayed a lack of consistent and robust reactivity on serological assays, and did not have detectable plasma viraemia, clinical follow-up was not deemed necessary, and was refused when offered.

Oral fluid EBOV IgG has been shown to have excellent concordance with plasma EBOV IgG, and the EBOV IgG capture assay has demonstrated excellent sensitivity and specificity [17]. Using this assay, seropositivity was found in 2.6% of 389 asymptomatic household contacts in Sierra Leone [17]. Using assays of different formats, 7.5% of 187 previously undiagnosed contacts were seropositive in Sierra Leone [19], and between 1% and 46% in previous outbreaks [11–13,15,16], although considerable concern has been expressed about the specificity of the assays used to generate these data [16,28]. In our study, samples were collected from individuals at various lengths of time after their return from West Africa, but no more than 2 y. The magnitude of the antibody response has been shown to decrease over time in some vaccine studies [29] but remained detectable several years after natural infection in previous outbreaks [30]. It is possible that the sensitivity of the assay was decreased by the OFS remaining at room temperature during postage; however, this seems unlikely as antibody concentrations in oral fluid have demonstrated stability at various temperatures for up to 7 d when tested using capture assays similar to that used in our study [31].

We demonstrate no evidence of infection with EBOV in individuals who were considered to have had a risk of transmission, or in returnees who had febrile or diarrhoeal symptoms during the potential incubation time for EBOV. A worrying proportion of responders had had direct physical contact with confirmed or suspected EVD patients, or near miss or exposure events inside the red zone, many of whom met similar exposure risk criteria to those administered favipiravir as post-exposure prophylaxis [25]. None of the returnees reported having been medically evacuated or having received post-exposure prophylaxis, but this was not asked about directly.

Reporting an event classified as very low, low, intermediate, or high risk for EBOV transmission was associated with performing a clinical role rather than a laboratory role. Broken vials and laboratory PPE breaches in Ebola laboratories have been described [32]. However, laboratory work may allow greater risk prediction and protocol-guided management than working with patients, who may present with confusion during the course of EVD [6]. Regular debriefing after work in the clinical red zone or laboratory and blame-free reporting of near misses should be part of routine practice in EMT response work. Together with the further development and publication of tools through which exposure risk can be graded, staff should be given realistic advice about their level of risk to reduce confusion and avoid anxiety, while allowing those with more than minimal risk to be identified. Responses from returnees who participated in this study could be used to inform ETC design and workforce planning to prevent potential EBOV exposure. For example, reports of staff experiencing skin lacerations from broken glass vials in the red zone should lead to consideration of a complete ban on the use of glass vials in this area. Reports of dislodged or torn PPE should lead to further testing of the robustness and fit of PPE suits, careful design of ETCs so that suits are not torn by catching on doorways or corners, and confirmation that protocols exist in each ETC for the management of PPE failure whilst in the red zone.

Performing a clinical role (nurses/doctors/paramedics) was strongly associated with describing an illness, compared to laboratory work or undertaking other roles. Clinical staff wore PPE for the majority of clinical interactions, minimising the risk of acquiring any infection from EVD patients. However, caring for unwell colleagues or mixing more with West African national staff may have increased this group’s exposure to transmissible pathogens to which they were immune-naïve, such as upper respiratory tract viral infections. It is concerning that a high proportion of returnees who experienced a febrile or diarrhoeal illness whilst within West Africa or within a month of return were not tested with PCR for EBOV at the time.

Being tested for EBOV infection was more strongly associated with experiencing an illness while in the UK compared to while still in West Africa. Illness in West Africa may have resulted in admission to a treatment centre where other confirmed EVD patients were being cared for, or medical evacuation to the UK. Although these would have been alarming for the individual and costly for the organisation or country of origin, earlier presentation with illness has been associated with improved survival [33,34]. The policies of NGOs, where available, were reviewed by the authors and frequently stated that EBOV should be tested for “after a period of observation” and if symptoms have “not resolved or progressed”. The pragmatic nature of these policies should be viewed in the context of the anxiety induced by testing, as well as potential exposure whilst inside a testing facility [35]. This study provides a reassuring lack of evidence of EBOV infection in individuals who had not previously been tested for EBOV, but who had had an illness consistent with mildly symptomatic EVD. Review, critique, and agreed standardisation of protocols for the management of illness in nationally and internationally recruited staff during outbreaks should be instigated.

This study included 268 individuals who returned OFSs. It was not a random sample, and it is possible that those who knew of possible exposures or who had had symptoms were particularly keen to participate. PHE conducted routine screening of all individuals who returned to the UK from West Africa at the peak of the epidemic, between 14 October 2014 and 4 December 2015. During this time, 646 individuals were designated low risk of exposure (including WASH staff, epidemiologists, health advisors, and contact tracers) or high risk of exposure (including HCWs providing EVD patient care, morgue workers, and burial teams) [36]. The use of oral fluid for testing, along with the use of a self-sampling device, is likely to have increased response rates and participation in our study [37]; it avoided the discomfort and inconvenience of blood sampling, and improved logistics for sample collection, given the geographical spread of returnees across the UK and Ireland.

By restricting the study to international staff, only 11 of whom had previous experience with filoviruses, we could evaluate the risk of contracting EBOV while working in PPE or otherwise responding to the epidemic, without the unquantifiable risk experienced by national HCWs who may have been exposed to infected family members, friends, and colleagues. Such contacts have been identified as the source of infection in the majority of HCW infections in Sierra Leone [38]. We were also able to evaluate potential transmission to individuals who worked in roles that did not predominantly involve direct work with patients with EVD, including epidemiologists, community engagement staff, researchers, and support staff such as those in finance or IT. Some of these individuals described contact with both positive and recovered cases, and considered that they had had near miss events or significant exposures. A clear correlation between level of exposure to EVD cases and their body fluids and the risk of acquiring EVD has been documented previously [17,39]. It is possible that potential transmission events or levels of risk were not captured in this study, particularly around variation in the eye protection component of PPE; EBOV had been detected in lung tissue [40], and transmission has been documented in individuals who did not recall physical contact with a confirmed case from whom they acquired infection [41].

Reporting a previously undisclosed illness during the incubation period for EVD, or not immediately reporting a near miss event, may be met with criticism or even professional disciplinary review [42]. Nevertheless, the returnees who participated in this study seemed willing to report these events. This was helped by avoiding face-to-face interaction through the use of an online consent form and questionnaire—a method that has been previously demonstrated to increase reporting of socially undesirable responses [43]—and by explicitly stating in the questionnaire that we would not identify specific treatment facilities or NGOs.

### Conclusion

The vast majority of international responders to the EVD epidemic who participated in this study had no evidence of EBOV infection. This should provide additional evidence in support of the effectiveness of ETC infection prevention and control procedures and of PPE. In view of the lack of evidence of infection in spite of the frequency of symptoms consistent with EVD (fever and diarrhoea), and the disconcerting level of under-testing at the time, consideration should be given to revising and standardising policies for management of illness. The level of concern expressed by returnees about potential exposure to EBOV both inside and outside red zone areas should highlight areas for improved support for national and international staff, and a need to standardise protocols for assessment and management of exposures between organisations.

## Supporting information

S1 STROBE Checklist (DOC)

S1 Text EBOV IgG returnees protocol. (DOCX)

S2 Text Questionnaire for returning healthcare workers. (PDF)

## Abbreviations

DABA: double antigen bridging assay
EBOV: Ebola virus
EMT: emergency medical team
ETC: Ebola treatment centre
EVD: Ebola virus disease
HCW: healthcare worker
IQR: interquartile range
LSHTM: London School of Hygiene & Tropical Medicine
NGO: non-governmental organisation
NOD: normalised optical density
OD: optical density
OFS: oral fluid sample
PHE: Public Health England
PPE: personal protective equipment
WASH: water, sanitation, and hygiene
WHO: World Health Organization
