# Supplementary material for: Ebola exposure, illness experience, and Ebola antibody prevalence in international responders to the West African Ebola epidemic 2014–2016: A cross-sectional study
Source: PLoS Med. 2017 May 16;14(5):e1002300. doi: 10.1371/journal.pmed.1002300 (PMC5433702; doi:10.1371/journal.pmed.1002300)
Supplement: S1 Text — (DOCX) [file pmed.1002300.s002.docx]

SEROSURVEY PROTOCOL

Title: Measuring asymptomatic infection in those returning to the UK after responding to the Ebola Zaire outbreak in West Africa: a serological survey

**Principal Investigator:** Catherine Houlihan

London School of Hygiene and Tropical Medicine (LSHTM)

Keppel St, Bloomsbury, London WC1E 7HT

United Kingdom

Telephone: +44 (0) 7474727797

[catherine.houlihan@lshtm.ac.uk](mailto:catherine.houlihan@lshtm.ac.uk)

**Scientists:** Marc Baguelin, B.Sc., M.Sc., Ph.D.

London School of Hygiene and Tropical Medicine (LSHTM)

Department of Infectious Disease Epidemiology

Keppel St, Bloomsbury, London WC1E 7HT

United Kingdom

Telephone: +44 (0) 208 327 6429 [marc.baguelin@lshtm.ac.uk](mailto:marc.baguelin@lshtm.ac.uk)

David Moore

London School of Hygiene and Tropical Medicine (LSHTM)

Keppel St, Bloomsbury, London WC1E 7HT

United Kingdom

Judith Glynn, PhD FRCP, Prof

London School of Hygiene and Tropical Medicine (LSHTM)

Keppel St, Bloomsbury, London WC1E 7HT

United Kingdom

Catherine McGowan, PhD

London School of Hygiene and Tropical Medicine (LSHTM)

Keppel St, Bloomsbury, London WC1E 7HT

United Kingdom

**Version number: 3 Date: 20-06-2016**

Table of Contents

[SEROSURVEY PROTOCOL 1](#_Toc475723565)

[Title: Measuring asymptomatic infection in those returning to the UK after responding to the Ebola Zaire outbreak in West Africa: a serological survey 1](#_Toc475723566)

[Introduction 2](#_Toc475723567)

[Background and Rationale 3](#_Toc475723568)

[Research Question 5](#_Toc475723569)

[Methods 7](#_Toc475723570)

[Type of Study 7](#_Toc475723571)

[Research Design 7](#_Toc475723572)

[Setting 7](#_Toc475723573)

[Population and Sample, sampling, selection criteria and size 7](#_Toc475723574)

[Method(s) of Data Collection and laboratory analysis 7](#_Toc475723575)

[Method(s) of Data Analysis 8](#_Toc475723576)

[Data storage and statistical package (if appropriate) 8](#_Toc475723577)

[Follow up of seropositive participants 8](#_Toc475723578)

[Infection prevention and control 8](#_Toc475723579)

[Compensation of participants 8](#_Toc475723580)

[Ethical Issues and Rigour 9](#_Toc475723581)

[Reference List 9](#_Toc475723582)

[Appendices 11](#_Toc475723583)

[Appendix 1 Consent form for returning health care workers 11](#_Toc475723584)

[Appendix 2 Consent form for control subjects 14](#_Toc475723585)

## Introduction

Ebola virus disease (EVD) is a severe infection caused by *Ebolavirus*, a Filo virus known to infect both humans and animals such as bats and non-human primates. The current EVD outbreak in West Africa has shaken the global public health community worldwide. One of the key features of the epidemic has been the high toll of healthcare workers, particularly at the beginning of the outbreak.

The proposed seroprevalence study aims to ascertain the proportion of individuals infected asymptomatically in a population of health care workers and researchers returning from the current outbreak in West Africa. It is unknown how frequently asymptomatic infections with Ebola occur. In previous outbreaks serological evidence of infection has been found in those without symptoms, but the specificity of these results has been uncertain, especially as they have been measured in populations with possible background exposure to other Filoviruses or non-outbreak exposure to Ebola. In a large survey in Gabon the prevalence of antibodies was similar in villages with and without known Ebola outbreaks (Becquart et al., 2010). Expatriate health workers are unlikely to have had such previous exposures so positive serology would suggest infection with Ebola.

Knowing the proportion of asymptomatic infections is essential for predicting the course of an epidemic. If a proportion of the population becomes immune without overt disease it would limit the spread of this and future epidemics.

The level of asymptomatic infection in healthcare workers is currently unknown. The identification of any asymptomatic Ebola infections may allow us to identify mechanisms involved in the transmission of the infection in a healthcare setting. It will enable us to put a new light on questions such as the possibility of paucisymptomatic/asymptomatic infection in this outbreak, links between level of exposure and probability of antibody response and infection. A lack of any asymptomatic infections in returnees may provide some assurance to those considering deployment during this or subsequent Ebola outbreaks.

To this aim, the investigators will analyse the presence of anti-Ebola antibodies by enzyme immunosorbent assay (EIA) in saliva among volunteer healthcare workers and researchers who have returned from participating in the response to the current Ebola outbreak in West Africa (2014/5). Individuals will be contacted on-line and provided with information about the study. Consent will also be requested on-line, and an on-line questionnaire will be sent to each consenting individual. The questionnaire will contain questions on activities undertaken in West Africa, and any recognized exposure. A kit and instructions for self-collection of oral fluid samples will be posted to consenting, eligible individuals with return postage (to PHE Collindale) paid. The presence of antibodies will be determined using an assay developed by Public Health England that is currently being used to examine presence of antibodies of contacts of cases of Ebola in Sierra Leone. Public Health England have additionally developed an in-house ELISA for IgG to Zaire Ebola virus. A commercial assay for IgG to GP and NP has also been developed by Alpha Diagnostics, and is being used in current vaccine studies (Rampling et al., 2015). One of these tests will be used to confirm samples that test positive using the saliva EIA. If alternative tests with higher specificity and/or sensitivity for previous infection with Ebola virus become available, these may be used in this study. Individuals with high antibody titres may be followed up with a further interview and, if they agree, a further sample will be requested 3 months after the first sample.

## Background and Rationale

Mounting evidence suggests that many Ebola infections may be asymptomatic (Becquart et al., 2010; Bertherat, Renaut, Nabias, Dubreuil, & Georges-Courbot, 1999; Busico et al., 1999; Gonzalez, Nakoune, Slenczka, Vidal, & Morvan, 2000; Heffernan et al., 2005; Heymann et al., 1980; E. M. Leroy et al., 2000). Asymptomatic individuals are assumed not to be able to transmit the infection. However, they could be protected against disease.

The Centres for Disease Control and Prevention Department of Health and Human Services (USA) Ebola Information Packet for International Laboratories, written in 2014, depicts the Ebola infection timeline (Figure 1). The viremia is shown to peak from three to ten days after the onset of symptoms and at this time the viral genome can be detected through RT-PCR. IgG antibodies, the target of our study, develop from day ten after symptom onset and are detected by ELISA from about day 17. IgG antibodies to Ebola Zaire have been detected more than 11 years after infection, making them excellent markers for infection (Wauquier, Becquart, Gasquet, & Leroy, 2009). In asymptomatic infection in those with no previous IgG antibodies to Ebola, IgM developed 10 days after last contact with a potentially infectious source, and IgG developed 7 days later (E. Leroy et al., 2000). During the current outbreak of Ebola in Sierra Leone, oral fluid EIA for Ebola antibodies has been compared to serum EIA and found to be equivalent (Prof Richard Tedder, personal communication).


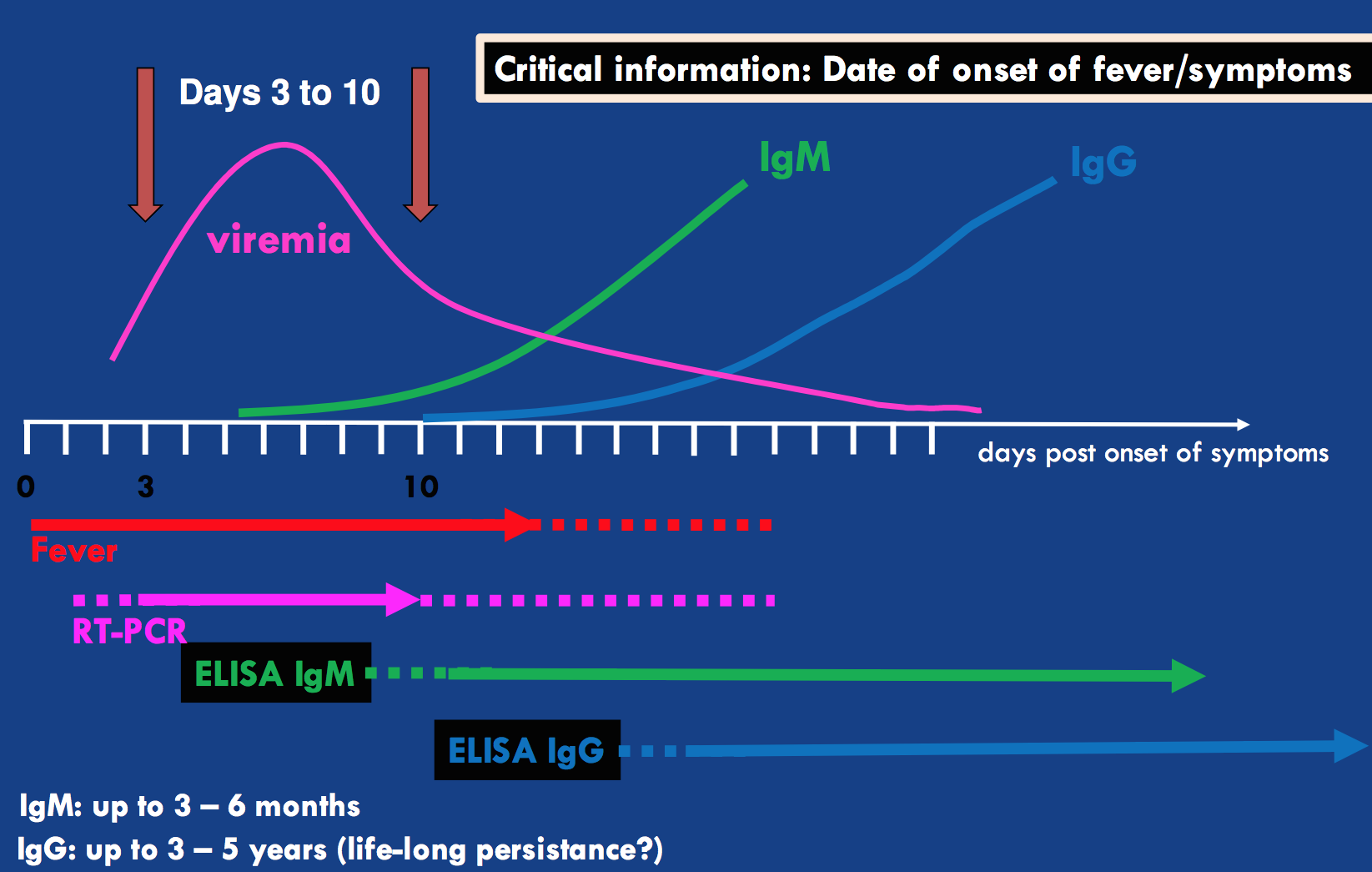


Figure 1. Timeline of the markers of Ebola infection. *Ebola Information Packet for International Laboratories Version 1.0*, 2014. Centres for Disease Control and Prevention Department of Health and Human Services (USA).

To our knowledge, no study has investigated the presence of EVD antibodies in those returning from fieldwork in West Africa during the current outbreak. The infection of some healthcare workers by the virus suggests that exposure exists at least at low level. Knowledge of the extent to which health care workers are exposed to the virus and how this is linked to infection is currently missing.

## Research Question

The proportion of healthcare workers and researchers returning from West Africa with positive antibody titres is unknown. If a proportion of returning healthcare workers have positive antibody to Ebola virus, we will explore the link between these antibodies and any potential exposure and presence of any symptoms. We will also explore the persistence of these antibodies after a period of 3 months.

The research question was assessed under the PICOT framework as follows:

1. Population:

Participants will include individuals who have returned from West Africa as part of the international response to the Ebola outbreak. Individuals who have not travelled to any of the countries where Ebola (of any type) has been acquired or diagnosed will be included in order to estimate the specificity of the test used during this study.

1. Comparison:

We will sample healthcare workers and researchers who have worked directly with patients within treatment centres, holding centres or community care centres (called ‘red zones’), those who have not worked within these areas but have worked in West Africa as part of the international response (those who only worked in areas called ‘green zones’), and laboratory workers. This means that we should get very distinct groups and a wide range of exposures, allowing comparison of different antibody responses.

Little data exist on specificity of serology tests for Ebola. Therefore, up to 100 individuals who have never travelled to any of the countries where Ebola (of any type) has been acquired or diagnosed, will additionally be tested in order to estimate the specificity of the test used during this study.

1. Outcomes:

(a) Previous EVD infection, determined by presence of antibody, (b) link between antibody and exposure to Ebola and finally

1. Timeline:

| **Major achievement or outcome** | **Activity** | **Start**  **date** | **End**  **date** |
| --- | --- | --- | --- |
| Ethical approval (LSHTM) | Submit application for ethical approval at LSHTM | 25^th^ March | 5^th^ April |
| Amendment | Submit ammendments | 31^st^ August |  |
| Consent and questionnaire completion | On-line consent sent, followed by on-line questionnaire to those consenting. | December 2015 | May 2016 |
| Sample collection | Postal of oral sample collection device | February 2016 | June 2016 |
| Sample testing | Laboratory analysis using antibodies to Ebola at PHE Collindale with confirmation of positive samples using PHE or Alpha Diagnostics ELISA. | May 2016 | June 2016 |
| Further follow-up interview of individuals with positive serology | Further interview of those with evidence of previous infection with Zaire Ebola virus. | June 2016 | June 2016 |
| Analysis | Statistical analysis | July 2016 | July 2016 |
| Dissemination | Initial communication of results and manuscript preparation | August 2016 | December 2016 |

## Methods

### Type of Study

Observational study.

### Research Design

This study aims to establish the proportion of returning healthcare workers with detectable antibody to *Ebolavirus Zaire* virus.

### Setting

Consent forms and questionnaires will be completed on-line. Oral fluid samples will be self-collected by consenting individuals and posted to the laboratory at Public Health England, Collindale. Data analysis and write up will be performed at the London School of Hygiene and Tropical Medicine, London.

### Population and Sample, sampling, selection criteria and size

This study aims to establish whether there have been any asymptomatic *Ebolavirus Zaire* infections in individuals returning from the current outbreak in West Africa. As the number of returning healthcare workers and researchers is small, we will attempt to test all returnees who agree to enroll in the study. The total numbers of healthcare workers and researchers returning from West Africa to the UK is estimated to be around 2,000-3,000, of whom 800-1,000 are expected to be contacted and agree to participate. Participants will be identified by PHE or from existing lists of agencies and through snow-ball sampling. All participants who have returned to the UK after working in West Africa during the current Ebola outbreak will be eligible for consent and questionnaire completion. Sampling will only take place after the individual has been in the UK for >21 days. Little data exist on specificity of serology tests for Ebola. Therefore, up to 100 individuals who have never travelled to any of the countries where Ebola (of any type) has been acquired or diagnosed, will additionally be tested in order to estimate the specificity of the test used during this study.

### Method(s) of Data Collection and laboratory analysis

Consent will be requested via an on-line form. Separate consent will be collected for returning health care workers (Appendix 1) and controls (Appendix 2). After an individual has consented to participate in the study, they will be asked to complete an electronic questionnaire. Separate questionnaires for returning health care workers (Appendix 3) and controls (Appendix 4) are attached. Equipment and instructions will be posted to each consenting participant in order that they self-collect an oral fluid sample. Pre-paid envelopes will be provided in order that the sample can be returned by post to PHE Collindale. Individuals who test positive in oral fluid will be requested to provide a sample of venous blood for confirmation. Approximately 10ml of blood will be drawn from each of these individuals via venipuncture by phlebotomy-trained personnel. All samples will be analysed at PHE Collindale according to PHE or commercial assay protocols .

### Method(s) of Data Analysis

Laboratory data will be linked with the questionnaire data and analysis of risk factors for antibodies to Ebola will be explored.

### Data storage and statistical package (if appropriate)

Only Dr. Catherine Houlihan and Dr. Catherine McGowan will have access to the data with patient identifiers. These will be stored under password protection, including during travel. All data with patient identifiers will be stored within the LSHTM network. Once the datasets have been linked, patient identifiers will be anonymised and replaced by a unique patient ID. Data will subsequently be analysed in this form.

Remaining oral fluid or serum from the study will be stored at the LSHTM ITD LAB 465, THPB1, Clinical Research Unit, Lab 246, Samples Archive and may be used in future projects as long as these projects have been granted permission by the Ethics Committee and consent from the participant has been obtained.

### Follow up of seropositive participants

Separate from the study, all individuals who test positive on oral fluid and blood serology will be invited for a clinical review at the Hospital for Tropical Diseases with one of Professor David Mabey, Professor David Moore or Professor Ron Behrens or at the Royal Free Hospital with Dr Mike Jacobs.

### Infection prevention and control

Individuals participating in the study will have returned from the field for more than 21 days and will not have presented symptoms of Ebola. Therefore no additional protective measure specific to Ebola will be required in order to collect saliva or blood samples. Standard procedures for blood sample collection will be used by the person trained in phlebotomy using gloves, tourniquet, alcohol swab, vacutainer adaptor and needle. One set of equipment will be used per patient and sharps will be safely disposed of.

### Compensation of participants

No compensation will be offered to participants. The results will be given to them after the testing if wished. The mechanism for communication of results will be established during on-line questionnaire completion.

## Ethical Issues and Rigour

This study will require ethical approval from the LSHTM because it involves the collection and handling of human samples and data with personal identifiers.

The main ethical concerns that should be taken into account are:

1. Informed consent:

Participants should be informed of

- - - The purpose of the study
    - Why they have been approached
    - That they are free to decline taking part in the study
    - That they are free to withdraw from the study at any time
    - What will happen if they decide to take part
    - The risks and benefits of taking part
    - That the confidentiality of their information will be respected
    - What will be done with the findings
    - That leftover samples may be used in future LSHTM studies approved by the ethics committee

They should confirm they understand this information and were given the opportunity to ask questions and confirm agreement to provide oral samples and to have a blood sample taken if necessary. Participants will be requested to complete the consent form online. They should consent free of any pressure (see Appendix 1 & 2).

1. Confidentiality:

The management of patient identifiers is outlined above and is carried out in accordance with information governance regulations.

1. Infection control precautions:

Appropriate equipment will be provided for safe sample collection and laboratory work. Samples are not considered infectious for Ebola Virus since they will be collected and tested once the participant has not been exposed for more than the recognized incubation period (21 days).

1. Compensation of participants: None
2. Publication policy:

All data without patient identifiers may be published in open access.

1. Training:

All researchers have received appropriate ethical training.

## Reference List

Becquart, P., Wauquier, N., Mahlakõiv, T., Nkoghe, D., Padilla, C., Souris, M., … Leroy, E. M. (2010). High prevalence of both humoral and cellular immunity to Zaire ebolavirus among rural populations in Gabon. *PloS One*, *5*(2), e9126. doi:10.1371/journal.pone.0009126

Bertherat, E., Renaut, A., Nabias, R., Dubreuil, G., & Georges-Courbot, M. C. (1999). Leptospirosis and Ebola virus infection in five gold-panning villages in northeastern Gabon. *The American Journal of Tropical Medicine and Hygiene*, *60*(4), 610–5. Retrieved from http://www.ncbi.nlm.nih.gov/pubmed/10348236

Busico, K. M., Marshall, K. L., Ksiazek, T. G., Roels, T. H., Fleerackers, Y., Feldmann, H., … Peters, C. J. (1999). Prevalence of IgG antibodies to Ebola virus in individuals during an Ebola outbreak, Democratic Republic of the Congo, 1995. *The Journal of Infectious Diseases*, *179 Suppl* (Supplement_1), S102–7. doi:10.1086/514309

Gonzalez, J. P., Nakoune, E., Slenczka, W., Vidal, P., & Morvan, J. M. (2000). Ebola and Marburg virus antibody prevalence in selected populations of the Central African Republic. *Microbes and Infection / Institut Pasteur*, *2*(1), 39–44. Retrieved from http://www.ncbi.nlm.nih.gov/pubmed/10717539

Heffernan, R. T., Pambo, B., Hatchett, R. J., Leman, P. A., Swanepoel, R., & Ryder, R. W. (2005). Low seroprevalence of IgG antibodies to Ebola virus in an epidemic zone: Ogooué-Ivindo region, Northeastern Gabon, 1997. *The Journal of Infectious Diseases*, *191*(6), 964–8. doi:10.1086/427994

Heymann, D. L., Weisfeld, J. S., Webb, P. A., Johnson, K. M., Cairns, T., & Berquist, H. (1980). Ebola Hemorrhagic Fever: Tandala, Zaire, 1977-1978. *Journal of Infectious Diseases*, *142*(3), 372–376. doi:10.1093/infdis/142.3.372

Ksiazek, T. G., West, C. P., Rollin, P. E., Jahrling, P. B., & Peters, C. J. (1999). ELISA for the detection of antibodies to Ebola viruses. *The Journal of Infectious Diseases*, *179 Suppl* (Supplement_1), S192–8. doi:10.1086/514313

Leroy, E., Baize, S., Volchkov, V., Fisher-Hoch, S., Georges-Courbot, M.-C., Lansoud-Soukate, J., … McCormick, J. (2000). Human asymptomatic Ebola infection and strong inflammatory response. *The Lancet*, *355*(9222), 2210–2215. doi:10.1016/S0140-6736(00)02405-3

Leroy, E. M., Baize, S., Volchkov, V. E., Fisher-Hoch, S. P., Georges-Courbot, M. C., Lansoud-Soukate, J., … Georges, A. J. (2000). Human asymptomatic Ebola infection and strong inflammatory response. *Lancet*, *355*(9222), 2210–5. Retrieved from http://www.ncbi.nlm.nih.gov/pubmed/10881895

Rampling T, Ewer K, Bowyer G, Wright D, Imoukhuede EB, Payne R, Hartnell F, Gibani M, Bliss C, …Hill, A. (2015). A Monovalent Chimpanzee Adenovirus Ebola Vaccine - Preliminary Report. *NEJM.* DOI:10.1056/NEJMoa1411627

Wauquier, N., Becquart, P., Gasquet, C., & Leroy, E. M. (2009). Immunoglobulin G in Ebola outbreak survivors, Gabon. *Emerging Infectious Diseases*, *15*(7), 1136–7. doi:10.3201/eid1507.090402

## Appendices

### Appendix 1 Consent form for returning health care workers

**Measuring asymptomatic infection in health care workers returning from the Ebola Zaire outbreak in West Africa: a serological survey.**

Background

Ebola virus disease (EVD) is a severe infection caused by *Ebolavirus*, a *Filoviridae* virus known to infect both humans and animals such as bats and non-human primates. The current EVD outbreak in West Africa has shaken the global public health community worldwide. One of the key features of the epidemic has been the high toll of healthcare workers, particularly at the beginning of the outbreak.

You are being asked to participate in a study coordinated by the London School of Hygiene and Tropical Medicine (LSHTM). This study will examine the presence of antibodies to Ebola in health care workers and researchers who have returned from West Africa after responding to the Ebola epidemic.

The identification of any asymptomatic Ebola infections may allow us to identify mechanisms involved in the transmission of the disease in a healthcare setting. A lack of Ebola infection in returnees who did not develop the disease will provide some assurance to those considering deployment during this or subsequent Ebola outbreaks.

The detection of antibodies to Ebola does not mean that an individual was definitely infected with Ebola. False positive results may occur with any serology (antibody) test and we will be evaluating the test during this study. If you are found to have antibodies to Ebola in the first test (oral fluid), you will be requested to provide a second sample (of blood). If both tests show that you have antibodies to Ebola, it may still be possible that this is a false positive result, or, it may indicate that you were infected with Ebola at some point. This does not indicate that you are infectious (i.e. might pass the infection to others), nor does it mean that you are immune (protected from infection with Ebola). If both tests are positive, you will be offered an appointment with an infectious diseases clinician so that you can ask questions about this. You will be invited to further interview and may be invited to participate in further separate research studies.

You are free to decline participation in this study, or to withdraw your participation at any time. If you choose to participate in this study, you will be asked to complete a questionnaire on-line, containing questions on what type of work and activities you were involved in in West Africa, whether you can remember any ‘near miss’ events which might have exposed you to Ebola, personal protective equipment used, and whether you had any illness in West Africa or since your return. We will then request a postal address. For some (but not all participants) we will send you the equipment and instructions to collect a sample of oral fluid. The package will include a pre-paid return envelope and instructions. If a further blood sample is required, we will contact you to arrange this. Blood samples will be a maximum of 10mls and will be collected by someone trained in phlebotomy (the taking of blood samples). If you wish to know the result of the tests, we will contact you via a method you specify (email, phone call, letter). If your sample is positive, we may request a further interview with you.

Your blood sample will be stored for 5 years, and may undergo further testing if, for example, a new test for Ebola becomes available. Your information will be fully anonymised and only Dr Catherine Houlihan and Dr Catherine McGowan will have access to your details.

I understand that if I accept to participate in this study, the following will happen:

- I will indicate my agreement at the end of this form
- I will be asked questions in an on-line form about my time in West Africa during the current Ebola outbreak including my role, activities and any near miss events or contact with patients or survivors with Ebola
- I may be asked to provide a oral fluid sample that will be tested for anti-bodies to Ebola
- I understand that I can refuse to participate in the study without giving any reason
- I will not receive any payment for my participation in this study
- The collection of any blood sample may result in discomfort at the site
- I will be able to access the results of the test which will indicate whether I have antibodies to Ebola
- If the tests show that I have antibodies to Ebola, this may indicate a false positive result, or may indicate that I was exposed to, or infected with Ebola at some point, and I have generated an antibody response to the virus.
- If I have antibodies to Ebola, there is no evidence that I am protected from catching Ebola and should consider myself at risk similarly to a person with no antibodies
- If I have antibodies to Ebola, I may be asked further questions and requested to provide a further sample
- If I have antibodies to Ebola in oral fluid and blood I will be offered a free clinical review appointment with a clinician at the Hospital for Tropical Diseases. This will be entirely voluntary and for my own personal health
- My oral fluid sample and/or blood sample will be stored at LSHTM and further testing on that sample may be performed if ethical approval is sought from LSHTM ethics board and my consent is obtained

I understand that if I have any questions on this study, or if there are things that I do not understand, I can contact the following people:

- Dr Catherine Houlihan London School of Hygiene and Tropical Medicine (LSHTM) Keppel St, Bloomsbury, London WC1E 7HT United Kingdom

Telephone: +44 (0) 7474727797

[catherine.houlihan@lshtm.ac.uk](mailto:catherine.houlihan@lshtm.ac.uk)

- Dr Catherine McGowan, London School of Hygiene and Tropical Medicine (LSHTM) Keppel St, Bloomsbury, London WC1E 7HT United Kingdom

Telephone: +44 (0)7990665658 catherine.mcgowan@lshtm.ac.uk

I understand that if I have any questions about my rights as a research study participant, I can contact the following person:

- Professor John Porter, Chair, Ethics Committee, London School of Hygiene & Tropical Medicine, England. Tel +44

**Confirmation**

- I confirm that I have read and understand the written information for this study. I voluntarily agree to participate in this study. If I have any additional questions, I will communicate them to the lead researcher via e-mail, phone call or in person.
- I understand that the lead researcher or any researcher who is part of this study, will not identify me by name in any manner, and in any reports using information obtained from this interview, and that my confidentiality as a participant in this study will remain secure
- I understand that I can leave the study at any time

### Appendix 2 Consent form for control subjects

###

**Measuring asymptomatic infection in health care workers returning from the Ebola Zaire outbreak in West Africa: a serological survey.**

Background

Ebola virus disease (EVD) is a severe infection caused by *Ebolavirus*, a *Filoviridae* virus known to infect both humans and animals such as bats and non-human primates. The current EVD outbreak in West Africa has shaken the global public health community worldwide. One of the key features of the epidemic has been the high toll of healthcare workers, particularly at the beginning of the outbreak.

You are being asked to participate in a study coordinated by the London School of Hygiene and Tropical Medicine (LSHTM). This study will examine the presence of antibodies to Ebola in health care workers and researchers who have returned from West Africa after responding to the Ebola epidemic.

You are being asked to participate in this study because we understand that you have **not** travelled to any country where transmission of Ebola is known to have occurred. So that we can establish the accuracy of the test we are using for antibodies to Ebola, we are interested in testing oral fluid samples from people who have **not** been exposed to the virus. If antibodies to Ebola are detected in your saliva, this will not mean that you have contracted Ebola in the past, nor will it mean that you are immune to Ebola in the future. It is likely that if your test result is positive for antibodies to Ebola, the result is a false positive, and the test is less reliable.

You are free to decline participation in this study, or to withdraw your participation at any time. If you choose to participate in this study, you will be asked to complete a brief questionnaire on-line about your demographic details and your health. We will then request a postal address and will send you the equipment and instructions to collect a sample of oral fluid. The package will include a pre-paid return envelope and instructions. Your sample will be stored for up to 5 years at LSHTM, and may undergo further testing if, for example, a new test for Ebola becomes available. Your information will be fully anonymised and only Dr Catherine Houlihan and Dr Marc Baguelin will have access to your details.

I understand that if I accept to participate in this study, the following will happen:

- I will indicate my agreement at the end of this form
- I will be asked questions in an on-line form about my demographics and health.
- I will be asked to provide an oral fluid sample that will be tested for anti-bodies to Ebola
- If I have antibodies to Ebola, this does not mean that I have been infected with Ebola at any point. Additionally, it does not mean that I have any natural protection against Ebola virus.
- I understand that I can refuse to participate in the study without giving any reason
- I will not receive any payment for my participation in this study
- My oral fluid sample will be stored at LSHTM and further testing on that sample may be performed if ethical approval is sought from LSHTM ethics board and my consent is obtained

I understand that if I have any questions on this study, or if there are things that I do not understand, I can contact the following people:

- Dr Catherine Houlihan London School of Hygiene and Tropical Medicine (LSHTM) Keppel St, Bloomsbury, London WC1E 7HT United Kingdom

Telephone: +44 (0) 7474727797

[catherine.houlihan@lshtm.ac.uk](mailto:catherine.houlihan@lshtm.ac.uk)

- Dr Catherine McGowan, London School of Hygiene and Tropical Medicine (LSHTM) Keppel St, Bloomsbury, London WC1E 7HT United Kingdom

Telephone: +44 (0)7990665658 catherine.mcgowan@lshtm.ac.uk

I understand that if I have any questions about my rights as a research study participant, I can contact the following person:

- Professor John Porter, Chair, Ethics Committee, London School of Hygiene & Tropical Medicine, England. Tel +44

**Confirmation**

- I confirm that I have read and understand the written information for this study. I voluntarily agree to participate in this study. If I have any additional questions, I will communicate them to the lead researcher via e-mail, phone call or in person.
- I understand that the lead researcher or any researcher who is part of this study, will not identify me by name in any manner, and in any reports using information obtained from this interview, and that my confidentiality as a participant in this study will remain secure
- I understand that I can leave the study at any time
