# Supplementary material for: Ebola exposure, illness experience, and Ebola antibody prevalence in international responders to the West African Ebola epidemic 2014–2016: A cross-sectional study
Source: PLoS Med. 2017 May 16;14(5):e1002300. doi: 10.1371/journal.pmed.1002300 (PMC5433702; doi:10.1371/journal.pmed.1002300)
Supplement: S2 Text — (PDF) [file pmed.1002300.s003.pdf]

Questionnaire for returning health care workers

Consent form

Before you complete the survey you will need to read an information sheet. Once you have read the information sheet you will be asked to consent to participate in the survey. Please read the following information closely.

Information sheet

Measuring asymptomatic infection in health care workers returning from the Ebola Zaire outbreak in West Africa: a serological survey

Ebola virus disease (EVD) is a severe infection caused by *Ebolavirus*, a *Filoviridae* virus known to infect both humans and animals such as bats and non-human primates. The current EVD outbreak in West Africa has shaken the global public health community worldwide. One of the key features of the epidemic has been the number of infections among healthcare workers, particularly at the beginning of the outbreak.

You are being asked to participate in a study coordinated by the London School of Hygiene & Tropical Medicine (LSHTM). This study will examine the presence of antibodies to Ebola in health care workers and researchers who have returned from West Africa after responding to the Ebola epidemic.

The identification of any asymptomatic Ebola infections may allow us to identify mechanisms involved in the transmission of the disease in a healthcare setting. A lack of Ebola infection in returnees who did not develop the disease will provide some assurance to those considering deployment during this or subsequent Ebola outbreaks.

The detection of antibodies to Ebola does not mean that an individual was definitely infected with Ebola. False positive results may occur with any serology (antibody) test and we will be evaluating the test during this study. If you are found to have antibodies to Ebola in the first test (oral fluid), you will be requested to provide a second sample (of blood). If both tests show that you have antibodies to Ebola, it may still be possible that this is a false positive result, or it may indicate that you were infected with Ebola at some point. This does not indicate that you are infectious (*i.e.* that you might pass the infection to others), nor does it mean that you are immune (*i.e.* protected from infection with Ebola). If both tests are positive, you will be offered an appointment with an infectious diseases clinician so that you can ask questions about this. You will be invited to attend a further interview and may be invited to participate in further separate research studies.

You are free to decline participation in this study, or to withdraw your participation at any time. We will send you the equipment and instructions to

collect a sample of oral fluid. The package will include a pre-paid return envelope and instructions. If a further blood sample is required, we will contact you to arrange this. Blood samples will be a maximum of 10mls and will be collected by someone trained in phlebotomy (the taking of blood samples). If you wish to know the result of the tests, we will contact you via a method you specify (email, phone call, letter).

Your sample(s) will be stored at the LSHTM for five years, and may undergo further testing if, for example, a new test for Ebola becomes available. Your information will be fully anonymised prior to analysis and publication of the results.

I understand that if I accept to participate in this study, the following will happen:

- I will indicate my agreement at the end of this form.
- I will be asked to provide a oral fluid sample that will be tested for anti-bodies to Ebola.
- I understand that I can refuse to participate in the study without giving any reason.
- I will not receive any payment for my participation in this study.
- The collection of any blood sample may result in discomfort at the site.
- I will be able to access the results of the test which will indicate whether I have antibodies to Ebola.
- If the tests show that I have antibodies to Ebola, this may indicate a false positive result, or may indicate that I was exposed to, or infected with Ebola at some point, and I have generated an antibody response to the virus.
- If I have antibodies to Ebola, there is no evidence that I am protected from catching Ebola and should consider myself at risk similarly to a person with no antibodies.
- If I have antibodies to Ebola, I may be asked further questions and requested to provide a further sample.
- If I have antibodies to Ebola in oral fluid and blood I will be offered a free clinical review appointment with a clinician at the Hospital for Tropical Diseases. This will be entirely voluntary and for my own personal health.
- My oral fluid sample and/or blood sample will be stored at LSHTM and further testing on that sample may be performed if ethical approval is sought from LSHTM ethics board and my consent is obtained.

I understand that if I have any questions on this study, or if there are things that I do not understand, I can contact the following people:

Dr Catherine Houlihan, London School of Hygiene and Tropical Medicine

Telephone: +44 (0) 74 7472 7797 Catherine.Houlihan@lshtm.ac.uk

Dr Catherine McGowan, London School of Hygiene and Tropical Medicine

Telephone: +44 (0)79 9066 5658 Catherine.McGowan@lshtm.ac.uk

I understand that if I have any questions about my rights as a research study participant, I can contact the following person:

Professor John Porter, Chair of the Ethics Committee, London School of Hygiene & Tropical Medicine.

Telephone: +44 (0) 20 7927 2298 John.Porter@lshtm.ac.uk

Confirmation

I confirm that I have read and understand the written information for this study. I voluntarily agree to participate in this study. If I have any additional questions, I will communicate them to the lead researcher via e-mail, phone call or in person. I understand that the lead researcher or any researcher who is part of this study, will not identify me by name in any manner, and in any reports using information obtained from this interview, and that my confidentiality as a participant in this study will remain secure I understand that I can leave the study at any time

- ◯ Yes, I understand the above and wish to participate in the study.
- ◯ No, I do not wish to participate in the study.

Ebola test

Have you ever had a test for Ebola virus? NOTE: If you have only ever been tested for antibodies please indicate NO since this question refers to tests for virus itself. If you are unsure, please contact us. ✱ *Required*

- ○ Yes
- ○ No

Test results

Have you ever tested positive for Ebola virus? ✱ *Required*

- Yes
- No

Contact details

Please indicate how we should contact you with your test results. ✱ *Required*

- ☐ E-mail
- ☐ Telephone
- ☐ Letter
- ☐ I do not want you to send me my results

Please enter the contact details you have specified above (i.e. e-mail address, phone number and/or postal address) - the contact details you provide will only be used for the communication of results. NOTE: If you have chosen not to receive your test results please write 'N/A' in the box below. ✱ *Required*

Please provide us with some basic information

Name ✱ *Required*

Gender ✱ *Required*

- Female
- Male

Date of birth ✱ *Required*

Dates need to be in the format 'DD/MM/YYYY', for example 27/03/1980.

(dd/mm/yyyy)

Please provide us with the postal address to which you would like us to send the sample text kit. ✱ *Required*

Have you ever received a filovirus vaccine (including Ebola vaccine)? ✱ *Required*

- Yes
- No

○ Unsure

Please tell us about your deployment

Where did you most recently work? (choose one) ✱ *Required*

- ○ Guinea
- ○ Liberia
- ○ Sierra Leone

Select all roles which you have undertaken in West Africa during the most recent epidemic: ✱ *Required*

- ☐ Burial team worker
- ☐ Community engagement staff
- ☐ Community contact tracing staff
- ☐ Engineer
- ☐ Epidemiologist
- ☐ Finance staff
- ☐ IT staff
- ☐ Laboratory technician/manager
- ☐ Logistician
- ☐ Management/operations staff
- ☐ Nurse (includes paramedic)
- ☐ Nutritionist
- ☐ Pharmacist
- ☐ Physician
- ☐ Researcher
- ☐ Social worker
- ☐ Trainer
- ☐ Water and sanitation (WASH) staff
- ☐ Other

If you selected Other, please specify:

Please tell us about your host organisation

With whom did you work? NOTE: Select the NGO you were working with if you were with UK Med. If you worked with more than one NGO, please select all. Please also note that no organisation will be identified without prior approval from that organisation, and that no individual will be personally identifiable.

Optional

- ☐ Emergencia
- ☐ GOAL
- ☐ Irish Medical Council (IMC)
- ☐ King's Sierra Leone Partnership (KSLP)
- ☐ London School of Hygiene & Tropical Medicine (LSHTM)
- ☐ Médecins Sans Frontières (MSF)
- ☐ Partners In Health (PIH)
- ☐ Red Cross
- ☐ Save the Children
- ☐ UK Ministry of Defence
- ☐ World Health Organization (WHO)
- ☐ Prefer not to say
- ☐ Other

If you selected Other, please specify:

____

Please list any other treatment centres/laboratories/holding centres you were based at or worked at during any Ebola response deployment.

At which treatment centre/laboratory/holding centre were you predominantly based during your deployment(s)?

Laboratory workers

Did you work in a laboratory during your deployment? ✱ *Required*

- Yes
- No

Estimate the total number of days you were working with samples of blood or other fluids from suspected or confirmed Ebola patients (during the whole epidemic) in the laboratory.

What was the most recent date you were working in the laboratory with blood or other body fluids from suspected or confirmed Ebola patients? NOTE: Please leave blank if you did not work with blood or other body fluids in the laboratory. *Optional*

Dates need to be in the format 'DD/MM/YYYY', for example 27/03/1980.

(dd/mm/yyyy)

Please tell us about your work in the Red Zone

Did you enter the Red Zone (the Red Zone is the area of the treatment centre/holding centre/community care centre where positive or suspected cases are found)? NOTE: The Red Zone does not include the laboratory. ✱ *Required*

- ○ Yes
- ○ No

Estimate the total number of days you went into the Red Zone (during the whole epidemic). *Optional*

What was the most recent date you were in the Red Zone? *Optional*

Dates need to be in the format 'DD/MM/YYYY', for example 27/03/1980.

(dd/mm/yyyy)

Please describe any other potentially risky contact you may have had outside the Red Zone.

Did you have any direct physical contact (*i.e.* shaking hands, embracing while NOT wearing PPE) with suspected/confirmed patients or their children outside of the Red Zone? Please include any episodes of direct contact with individuals who were unwell with a febrile illness but not considered suspect cases. NOTE: This does not include contact with survivors. ✱ *Required*

- Yes
- No
- Unsure

If yes (or unsure) to above please describe this in as much detail as possible including the number of times, type of contact, and month/year this occurred**.**

Did you have any physical contact (shaking hands, embrace etc) with patients who were ebola survivors? ✱ *Required*

- Yes
- No
- Unsure

If yes (or unsure) to above please describe this as much as possible including the number of times, type of contact and month/year this occurred,whether this

was part of an ETC discharge process and whether these were recent (less than one month recovered) or not recent survivors.

Please describe any incidents which might have resulted in an exposure.

Did you have any ‘near miss’ incidents (excluding previous page) or exposures? *e.g.* torn glove, needlestick, splashes, goggle/visor/facemask dislodged. ✱ *Required*

- Yes
- No
- Unsure

If yes (or unsure) to above please describe this as much as possible including the number of times, type of contact, and month/year this occurred.

Did you have a febrile illness either while in country or within a month of return? ✱ *Required*

- Yes
- No
- Unsure

If yes (or unsure) please please describe this as much as possible including the number of times, your symptoms, treatment, and approximate month/year this occurred.

Have you previously worked in viral haemorrhagic fever outbreaks? ✱ *Required*

- Yes
- No

Have you previously worked in a laboratory with Ebola virus/other filovirus? ✱ *Required*

- Yes
- No

Personal Protective Equipment (PPE)

Were you required to wear PPE while at work (please respond YES even if you were required to wear minimal PPE, *e.g.* only gloves). NOTE: If you only wore PPE while delivering or attending training then please respond NO. ✱ *Required*

- ○ Yes
- ○ No

What PPE were you provided with?

If you were provided with or used different PPE on different occasions, please check all that apply.

Gloves

- ☐ None
- ☐ Single pair of surgical gloves
- ☐ Double pair of surgical gloves
- ☐ Single pair of heavy duty gloves
- ☐ Surgical gloves underneath heavy duty gloves
- ☐ Triple gloves
- ☐ Other

If you selected Other, please specify:

Suit

- ☐ None
- ☐ Lightweight protective suit (without hood)
- ☐ Heavyweight protective suit (without hood)
- ☐ Lightweight protective suit and hood (either seperate or part of suit)
- ☐ Heavyweight protective suit and hood (either seperate or part of suit)
- ☐ Other

If you selected Other, please specify:

Eye protection

- [ ] None
- [ ] Goggles
- [ ] Face shield/visor
- [ ] Other

If you selected Other, please specify:

Respiratory protection

- [ ] None
- [ ] Surgical mask
- [ ] N95 (or similar) respirator
- [ ] N95 (or similar) respirator with surgical mask or hood mask on top
- [ ] Other

If you selected Other, please specify:

Please describe your experience removing PPE

With respect to PPE removal, please select from the following (which applies to the majority of your experience).

- PPE removal without any chlorine spray
- PPE removal with chlorine spray below waist only
- PPE removal with chlorine spray above and below waist

Who carried out your PPE removal, please select from the following (please choose based on the majority of your experiences)?

- PPE removal alone with no assistance
- PPE removal with assistance (physical or verbal)

Did you receive splash to eye/mouth during PPE removal?

- No
- Once or twice
- Several times
- Unsure

Did you have any concerns with the risk of exposure during your usual PPE removal steps?

- Yes
- No

If YES, please briefly describe your concerns.

Thank you for completing this survey!

Thank you for taking the time to complete this survey. If you have completed any section of this survey in error, or have any other feedback about the survey, please feel free to contact the data manager: Dr. Catherine McGowan (Catherine.McGowan@lshtm.ac.uk).

Once a sufficient number of people have completed the survey we will send out the test kits in the post.

Final reminder!!

As a final reminder - please forward the link to this survey to anyone you know who has returned to the UK after responding to the Ebola epidemic in West Africa.
